# Associations between biomarkers of bone and cartilage turnover, gender, pain categories and radiographic severity in knee osteoarthritis

**DOI:** 10.1186/s13075-019-1987-7

**Published:** 2019-09-03

**Authors:** Asger Reinstrup Bihlet, Inger Byrjalsen, Anne-Christine Bay-Jensen, Jeppe Ragnar Andersen, Claus Christiansen, Bente Juel Riis, Morten A. Karsdal

**Affiliations:** 1 Nordic Bioscience Clinical Development, Herlev Hovedgade 82, DK2730 Herlev, Denmark; 2 Nordic Bioscience Biomarkers and Research, Herlev Hovedgade 207, DK2730 Herlev, Denmark; 3 Nordic Bioscience A/S, Herlev Hovedgade 207, DK2730 Herlev, Denmark

**Keywords:** Osteoarthritis, Biomarkers, Bone, Cartilage, Radiography, Pain

## Abstract

**Background:**

Excessive cartilage degradation is a known characteristic of osteoarthritis (OA). Biochemical markers, such as uCTX-II, have been shown to be associated with disease severity, yet the tissue origin of CTX-II has been disputed. This analysis investigates the association between OA knee joints at different radiographic stages and pain categories with levels of uCTX-II and biomarkers of bone resorption and formation.

**Methods:**

Baseline data of two randomised clinical trials (NCT00486434 and NCT00704847) in patients with radiographic OA and presence of pain were analysed post hoc. A subgroup with available urine samples and evaluable radiographs for both knees (*N* = 1241) was analysed. Urine CTX-I, urine CTX-II and serum osteocalcin were analysed for associations with combined Kellgren-Lawrence (KL) scores, gender and pain for both knees to assess the contribution of joints at different stages.

**Results:**

Pain, BMI, age, gender and KL grade were all significantly associated with uCTX-II. The association between pain and CTX-II appeared to be driven by weight-bearing pain. The level of uCTX-II incrementally increased with higher radiographic severity of each knee. Levels of bone markers CTX-I and osteocalcin were both significantly associated with BMI and gender, but neither were associated with radiographic severity. Biomarker levels between male or female groups of identical KL scores were found to be higher in females compared to males in some but not all KL score groups.

**Conclusions:**

These results indicate that levels of uCTX-II are independently associated with radiographic severity of OA and pain intensity. CTX-II was associated with weight-bearing pain, but not non-weight-bearing pain, independent of co-variates. Bilateral OA knee joints appear to contribute to uCTX-II levels in an incremental manner according to radiographic severity of single joints. The data suggest that biomarker differences between genders should be taken into account when evaluating these markers in the context of structural features of OA.

## Background

Osteoarthritis (OA) is the most common arthritic disease, affecting more than 250 million people in the world [1]. The disease is characterised by excessive cartilage degradation, abnormal bone growth and sclerosis and synovial inflammation in a subset of patients [2–4]. Assessment of disease severity is based on clinical evaluations of pain, joint stiffness and limitations in physical function as well as radiographic assessment of osteophytes, bone sclerosis and joint-space narrowing, using the Kellgren and Lawrence grading scale [5]. The biochemical marker (biomarker) urine C-telopeptide of cross-linked collagen type II, uCTX-II, has been shown to be associated with OA disease severity [6–8], OA pain [9, 10] and possibly also as a marker of therapeutically derived structural modification in OA [11]. It was recently found to currently be the most suitable single biochemical marker for prediction of disease progression in OA in a consortium funded by the Foundation of National Institutes of Health (FNIH) [12]. Importantly, the level of uCTX-II has also been shown to be incrementally affected by one or multiple arthritic joints, yet hip OA appeared more closely associated with elevations in uCTX-II than knee, hand and facet joint OA [13]. Despite the aforementioned detailed analysis, data relating to the contribution of single or multiple OA joints to uCTX-II levels at more clearly defined radiographic stages of disease remain unclear, and data from large studies with established, painful OA of the knees on whether early- or late-stage disease contributes equally are not available. While several reports supporting the suitability of uCTX-II as a robust measure of cartilage degradation exist, its reported association with known biomarkers of bone turnover, including the bone resorption marker C-telopeptide of cross-linked collagen type I (CTX-I) in urine (uCTX-I) and the bone formation marker serum (s-)osteocalcin (N-MID), has challenged the notion that uCTX-II reflects cartilage degradation [14]. Other reports indicate that uCTX-II may reflect turnover of calcified cartilage [15]. Reports have found associations between uCTX-II and osteophyte area in painful knees, but not in knees with no pain, while associations found between uCTX-II and joint space width were similar in both knees with and without pain [16]. Another report found an association between elevated uCTX-II and symptomatic OA and speculates that uCTX-II, among others, may reflect early changes in cartilage accounting directly or indirectly for knee pain [10].

To further investigate the tissue origin and pathological relevance of these biomarkers, this report will assess the associations between pain, gender and radiographic severity of OA and uCTX-II, uCTX-I and s-osteocalcin.

## Patients and methods

### Study population

This is a post hoc, cross-sectional analysis of two, double-blinded, randomised, placebo-controlled and multicenter phase III clinical trials assessing the efficacy and safety of an oral formulation of 0.8 mg salmon calcitonin in patients with painful knee OA (NCT00486434 (trial 1) and NCT00704847 (trial 2)). The trials were conducted in accordance with the Helsinki Declaration and ICH GCP and were approved by all applicable Independent Review Boards, Ethics Committees and regulatory bodies. Each independent trial recruited patients aged 51–80 years with painful OA in the target knee, defined as a visual analogue score of ≥ 150 mm on the Western Ontario and McMaster Universities Osteoarthritis Index (WOMAC) pain sub-scale (500 mm being the maximum score). In study 2, patients scoring ≤ 150 mm on the pain sub-score were allowed to participate if they also scored ≥ 510 mm on the WOMAC function sub-scale (1700 mm being the maximum score). The radiographic inclusion criteria for target knees included Kellgren-Lawrence (KL) grade 2 or 3, and a joint space width (JSW) of ≥ 2.0 mm. A total of 2206 patients were recruited at 19 sites in 11 countries. Patients were followed for 2 years with regular clinic visits. Details regarding trial design and results are published elsewhere [17]. A single target knee was selected for each subject upon randomisation. A subgroup with available urine samples and evaluable radiographs for both knees (*N* = 1241) was analysed for this report. Participants were required to fast for 8 h before biomarker blood sampling was performed, but no restrictions on vitamin or dietary supplementation with potential relevance to the biomarkers were required.

### Radiographic evaluation

X-ray images of both knees using fixed flexion were obtained at the screening visit to assess the eligibility for study participation and to select the target knee. X-ray images were read by expert radiologists for JSW and KL grade. For the purpose of this post hoc analysis, radiographs acquired at the screening visit were used. Radiographic evaluation of non-knee joints was not performed in the context of the present trials.

### Evaluation of pain

Subject’s baseline pain intensity was measured using the pain sub-score of the WOMAC patient-reported outcome tool, consisting of five questions each scored on a visual analogue scale from 0 to 100 mm, where 100 mm is the worst pain imaginable. For data analysis in the current report, the WOMAC pain sub-score of each knee was normalised to 0–100, analysed as a continuous variable and for graphical depiction of associations with biomarkers, divided into tertiles for each knee, resulting in six groups ranging from mild pain in both knees to severe pain in both knees. The pain sub-scale records patient assessments in the following five situations/questions: (1) during walking on a flat surface, (2) using stairs (up or down), (3) at night while in bed, (4) sitting or lying and (5) while standing. Composites of the WOMAC pain sub-scale were constructed to detect associations between biomarkers and (A) pain experienced while the joint was weight-bearing, and/or the patient was active (questions 1, 2 and 5) or (B) pain experienced while idle and while the joint was free of mechanical load (questions 3 and 4).

### Biochemical marker assays

Assays for type II collagen degradation product uCTX-II, type I collagen degradation product uCTX-I and the mid protein form of s-osteocalcin (N-MID) were conducted on samples acquired from patients at baseline. N-MID was measured individually by the fully automated Elecsys® electro-chemiluminescent immunoassay analysers using the S-NMID osteocalcin assay (Roche Diagnostics, GmbH, Mannheim, Germany). Urinary CTX-I and CTX-II were determined using the Urine CrossLaps and Urine Cartilaps ELISAs (IDS Nordic, Herlev, Denmark). Urinary creatinine was measured by a routine chemistry method and used for calculation of creatinine-corrected urinary CTX-I and CTX-II concentrations.

### Statistical analyses

Univariate and multivariate regression analyses were performed using a general linear model ANOVA to evaluate potential associations among the parameters of age, the sum of WOMAC pain sub-score for both knees and as composites of weight-bearing (WB) pain or non-weight-bearing (NWB) pain, with each of the biomarkers. As the pain category WOMAC pain contains variables also included in WOMAC weight-bearing pain and WOMAC non-weight-bearing pain as described above, one multivariate model per each of these groups is performed. In the regression analyses, the biomarker values were logarithmically transformed to obtain normality. The parameter estimates and the standard error of estimate from the regression analyses are back-transformed in the presentation of the outcome of the regression analyses whereby the estimated effect and the confidence interval of the baseline characteristics will be given in the unit of percentage of the biomarker value. Parameters with a *p* value below 0.05 were defined as significantly associated with the given biomarker concentration.

Mean biomarker concentrations between KL grade groups and pain groups were compared using a one-way ANOVA for groups of all participants or separated by gender and adjusted for multiple comparisons by Tukey’s multiple comparison method, with an alpha of 0.05. Using an identical method, mean biomarker concentrations in groups of identical KL grades were also compared between males and females.

Correlation of biomarkers was evaluated using Spearman’s correlation in the total study population and in groups of males and females, respectively.

Statistical analyses were performed using SAS™ software (Cary, NC, USA).

## Results

Table 1 shows the demographic and clinical characteristics of the study population.

**Table 1:** Demographic characteristics

Demographic baseline characteristics	
Parameter	*N* = 1241
Sex, *n* (%)	
Male	399 (32.2)
Female	842 (67.8)
Age (years)	
Mean (SD)	64.6 (6.6)
Median (min, max)	64.5 (50.4–80.4)
Race, *n* (%)	
Caucasian	1110 (89.4)
Asian	130 (10.5)
Other	1 (0.1)
BMI, kg/m^2^	
Mean (SD)	28.9 (4.7)
WOMAC pain (0–100), target knee (SD)	47.5 (14.6)
WOMAC pain (0–100), non-target knee (SD)	35.7 (22.3)
Knee OA, *n* (%)	
Unilateral, early	44 (3.5)
KL 0/2	44 (3.5)
KL 0/1	0 (0)
Unilateral, late	1 (< 0.1)
KL 0/3	1 (< 0.1)
KL 0/4	0 (0)
Bilateral, early	851 (68.6)
KL 1/2	221 (17.8)
KL 2/2	630 (50.8)
Bilateral, early/late	250 (20.1)
KL 1/3	20 (1.6)
KL 1/4	0 (0)
KL 2/3	212 (17.1)
KL 2/4	18 (1.5)
Bilateral late/late	95 (7.7)
KL 3/3	80 (6.4)
KL 3/4	15 (1.2)
KL 4/4	0 (0)

*BMI* body mass index, *KL* Kellgren-Lawrence, *JSW* joint space width, *WOMAC* Western Ontario and McMaster Universities Osteoarthritis Index, *OA* osteoarthritis

Unilateral knee OA as defined in the current analyses was noted in 3.6% of patients in the study. 68.2% of patients had bilateral, early knee OA, 7.5% had bilateral late knee OA and 19.7% had bilateral knee OA of mixed stages. One patient had unilateral, late-stage knee OA (KL 0 and 3, respectively). The latter subject was excluded from analysis of differences between groups of radiographic stages as a single data point would not provide an analysable mean and standard deviation.

**Fig. 1:**
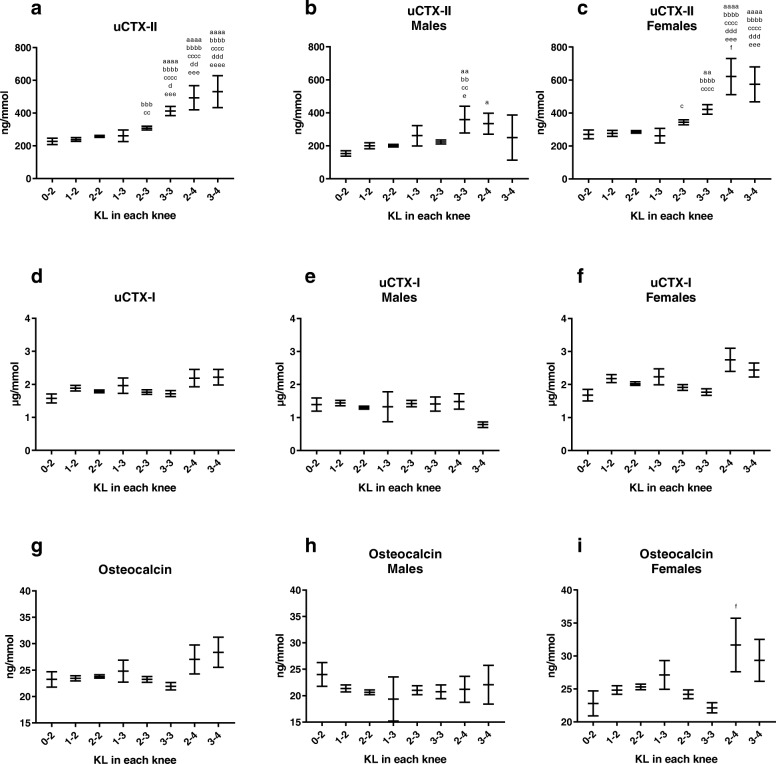
Association between Kellgren-Lawrence and biomarkers of bone and cartilage turnover. Association between geometric mean concentration of uCTX-II (creatinine corrected) (Fig. 1**a**-**c**), uCTX-I (creatinine corrected) (Fig. 1**d**-**f**) and serum osteocalcin (Fig. 1**g**-**i**)

As shown in Fig. 1, the mean uCTX-II concentration was significantly higher in patients with the bilateral knee combinations of KL grades 3 and 4, 3 and 3, and 2 and 4, as compared to KL grades of 0 and 2. In patients with at least one joint of KL 4, uCTX-II levels of more than double that of the group of KL 0 and 2 were measured, indicating a clear association between uCTX-II and radiographic severity of knee OA. Upon separation of the groups by gender, the findings were generally similar, although the magnitude of elevation in the higher KL groups was higher in females as compared with males.

No statistically significant differences between groups of KL grades for uCTX-I or osteocalcin were found; however, trends indicating higher levels of both markers were seen for groups of patients each with at least one knee with KL 4. The trends found for uCTX-I and osteocalcin appear to be driven by the female study population, as very little difference between radiographic grades was seen in males. The levels of uCTX-I, uCTX-II and osteocalcin were also found to be significantly higher in females than in males in groups of those with identical radiological scores (Table 2).

**Table 2:** Univariate pairwise comparisons of mean biomarker levels between males and females in groups of those with identical radiological scores

KL grade in each knee (*N*_male_/*N*_female_)	uCTX-II, mean, ng/mmol (SD)			uCTX-I, mean μg/mmol (SD)			Osteocalcin mean ng/mmol (SD)		
	Men	Women	*p* value for difference	Men	Women	*p* value for difference	Men	Women	*p* value for difference
*0–2* (16/28)	153.8 (67.1)	269.8 (139.3)	0.24	1.39 (0.80)	1.68 (0.94)	0.98	24.02 (8.94)	22.80 (9.99)	0.99
*1–2* (88/133)	*200.4 (167.3)*	*275.9 (241.6)*	*0.01*	*1.44 (0.77)*	*2.18 (1.39)*	*< 0.0001*	*21.37 (21.37)*	*24.83 (7.53)*	*0.01*
*2–2* (203/426)	*200.5 (111.5)*	*284.9 (161.7)*	*< 0.0001*	*1.30 (0.64)*	*2.03 (1.14)*	*< 0.0001*	*20.64 (6.44)*	*25.31 (8.83)*	*< 0.0001*
*1–3* (6/14)	261.3 (150.4)	261.9 (166.0)	> 0.99	1.33 (1.11)	2.23 (0.91)	0.43	19.36 (10.26)	27.13 (8.12)	0.30
*2–3* (63/149)	*223.8 (96.2)*	*343.9 (186.8)*	*< 0.0001*	*1.43 (0.76)*	*1.91 (1.08)*	*0.01*	21.04 (6.60)	24.18 (8.16)	0.06
*3–3* (11/69)	358.9 (267.3)	421.6 (245.0)	0.92	1.41 (0.70)	1.77 (0.84)	0.92	20.74 (4.37)	22.13 (6.37)	0.99
*2–4* (8/10)	*334.3 (179.8)*	*621.1 (347.2)*	*0.004*	1.49 (0.65)	2.75 (1.11)	0.07	*21.22 (6.94)*	*31.67 (12.85)*	*0.04*
*3–4* (2/13)	250.0 (193.7)	574.1 (383.4)	0.11	0.79 (0.12)	2.44 (0.76)	0.23	22.07 (5.19)	29.34 (11.46)	0.87

*KL* Kellgren-Lawrence. Statistically significant differences between groups are highlighted in italics

**Fig. 2:**
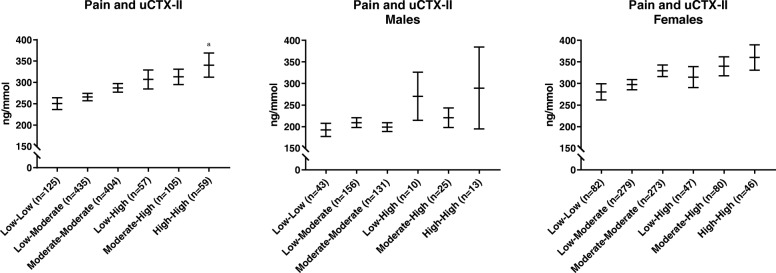
Association between WOMAC pain and the CTX-II biomarker of cartilage turnover. Association between geometric mean concentration of uCTX-II (creatinine corrected) and pain intensity in the full study population (*N* = 1241) and by gender (male: *N* = 399, female: *N* = 842). Groups of pain intensity consist of tertiles of WOMAC pain sub-score intensity of each knee (ranging from 0 to 100), resulting in six groups ranging from mild pain in both knees (“Low-Low”) to severe pain (“High-High”) in both knees. Error bars represent ± 1sem range. “a” *p* < 0.05 compared to the “Low-Low” group. WOMAC, Western Ontario and McMaster Universities Osteoarthritis Index

In multivariate analysis, age, BMI, female gender, pain and KL grade in either knee were all found to be significantly associated with uCTX-II. These associations remained when adjusting for co-variates in a multivariate analysis, but when analysing pain categories of weight-bearing and non-weight-bearing pain, only weight-bearing pain remained significantly associated with CTX-II. As shown in Table 3, a 10-point (on a 0–100 scale) increase in weight-bearing pain contributes to the uCTX-II level more than three times that of an additional 1 year of age or 1 kg/m^2^ of BMI. The association between pain and uCTX-II in the total study population and by gender is illustrated in Fig. 2.

**Table 3:** Estimated effect of baseline characteristics on uCTX-II biomarker level

Descriptive variable	Univariate		Multivariate		Multivariate		Multivariate	
	Estimate (95% CI) %	*p* value	Estimate (95% CI) %	*p* value	Estimate (95% CI) %	*p* value	Estimate (95% CI) %	*p* value
WOMAC pain (per 10 points)	5.5 [3.3;7.7]	*< 0.001*	2.2 [0.2;5.2]	*0.03*	–	–	–	–
WOMAC WB pain (per 10 points)	5.4 [3.2; 7.6]	*< 0.001*	–	–	2.2 [0.2;4.2]	*0.03*	–	–
WOMAC NWB pain (per 10 points)	3.1 [1.3;4.9]	*< 0.001*	–	–	–	–	1.3 [− 0.3;2.9]	0.11
Age (per year)	1.0 [0.4;1.6]	*< 0.001*	0.5 [0.1;0.9]	*0.02*	0.6 [0.0;1.2]	*0.02*	0.6 [0.0;1.2]	*0.01*
BMI (per 1 kg/m^2^)	1.2 [0.6;1.8]	*< 0.001*	0.8 [0.2;1.4]	*0.02*	0.7 [01;1.3]	*0.03*	0.8 [0.0;1.6]	*0.02*
Female sex	47.6 [38.0;57.2]	*< 0.001*	41.7 [32.7;50.7]	*< 0.001*	41.4 [32.4;50.4]	*< 0.001*	41.7 [32.7;50.7]	*< 0.001*
KL (per KL grade)	18.5 [14.8;22.2]	*< 0.001*	14.8 [11.0;18.5]	*< 0.001*	14.6 [10.9;18.3]	*< 0.001*	15.1 [11.4;18.8]	*< 0.001*

Effects of baseline body mass index, sex, the sum of Kellgren-Lawrence grade of the non-target and the target knee and WOMAC pain to the baseline uCTX-II value, in percent and in univariate and multivariate analyses. WOMAC pain and sub-categories are calculated as the sum of both knees, normalised to 0–100 and analysed as the sum of the five pain sub-score questions, and as composites of weight-bearing (WB) pain or non-weight-bearing (NWB) pain. As the category “WOMAC pain” contains variables also included in “WOMAC WB pain” and “WOMAC NWB pain”, one multivariate model per each of these groups is performed. The parameter estimates and the standard error of estimate from the regression analyses are back-transformed in the presentation of the outcome of the regression analyses whereby the estimated effect and the confidence interval of the baseline characteristics will be given in the unit of percentage of the biomarker value. *p* values < 0.05 were considered significant and highlighted in italics. *WOMAC* Western Ontario and McMaster Universities Osteoarthritis Index, *BMI* body mass index, *KL* Kellgren-Lawrence, *uCTX-II* urine C-telopeptide of cross-linked collagen type II

BMI and female gender were found to be significantly associated with uCTX-I (Table 4) and osteocalcin, and in addition, weight-bearing pain was weakly, negatively associated with osteocalcin independently of co-variates, as shown in Table 5. Female gender contributes greatly to the level of particularly uCTX-I and uCTX-II, for which female gender increases the biomarker level nearly 50% compared to that in males.

**Table 4:** Estimated effect of baseline characteristics on uCTX-I biomarker level

Descriptive variable	Univariate		Multivariate		Multivariate		Multivariate	
	Estimate (95% CI) %	*p* value	Estimate (95% CI) %	*p* value	Estimate (95% CI) %	*p* value	Estimate (95% CI) %	*p* value
WOMAC pain (per 10 points)	1.6 [− 0.6;3.8]	0.14	0.6 [− 1.4;2.6]	0.56	–	–	–	–
WOMAC WB pain (per 10 points)	0.2 [− 1.8;2.2]	0.83	–	–	− 0.3 [− 2.2;1.7]	0.77	–	–
WOMAC NWB pain (per 10 points)	2.1 [0.3;3.9]	*0.02*	–	–	–	–	1.1 [− 0.7;2.9]	0.19
Age (per year)	0.0 [− 0.4;0.4]	0.89	− 0.4 [− 0.8;− 0.0]	0.12	− 0.3 [− 0.9;− 0.3]	0.13	− 0.4 [− 0.8;− 0.0]	0.13
BMI (per 1 kg/m^2^)	− 3.0 [− 3.6;− 2.4]	*< 0.001*	− 3.2 [− 3.8;− 2.6]	*< 0.001*	− 3.2 [− 4.0;− 2.4]	*0.03*	− 3.2 [− 3.8;− 2.6]	*< 0.001*
Female sex	45.4 [36.0;54.8]	*< 0.001*	47.5 [38.1;56.9]	*< 0.001*	47.9 [38.5;57.3]	*< 0.001*	47.2 [37.8;56.6]	*< 0.001*
KL (per KL grade)	1.2 [− 2.3;4.7]	0.50	1.4 [− 1.9;4.7]	0.42	1.6 [− 1.7;4.9]	0.34	1.3 [− 2.0;4.6]	0.44

Effects of baseline body mass index, sex, the sum of Kellgren-Lawrence grade of the non-target and the target knee and WOMAC pain to the baseline uCTX-I value, in percent and in univariate and multivariate analyses. WOMAC pain and sub-categories are calculated as the sum of both knees, normalised to 0–100 and analysed as the sum of the five pain sub-score questions, and as composites of weight-bearing (WB) pain or non-weight-bearing (NWB) pain. As the category “WOMAC pain” contains variables also included in “WOMAC WB pain” and “WOMAC NWB pain”, one multivariate model per each of these groups is performed. The parameter estimates and the standard error of estimate from the regression analyses are back-transformed in the presentation of the outcome of the regression analyses whereby the estimated effect and the confidence interval of the baseline characteristics will be given in the unit of percentage of the biomarker value. *p* values < 0.05 were considered significant and highlighted in italics. *WOMAC* Western Ontario and McMaster Universities Osteoarthritis Index, *BMI* body mass index, *KL* Kellgren-Lawrence, *uCTX-I* urine C-telopeptide of cross-linked collagen type I

**Table 5:** Estimated effect of baseline characteristics on osteocalcin biomarker level

Descriptive variable	Univariate		Multivariate		Multivariate		Multivariate	
	Estimate (95% CI) %	*p* value	Estimate (95% CI) %	*p* value	Estimate (95% CI) %	*p* value	Estimate (95% CI) %	*p* value
WOMAC pain (per 10 points)	− 0.3 [− 1.5;0.9]	0.64	− 0.7 [− 1.9;− 0.5]	0.24	–	–	–	–
WOMAC WB pain (per 10 points)	− 1.1 [− 2.2;− 0.1]	0.06	–	–	− 1.3 [− 2.4;− 0.1]	*0.02*	–	–
WOMAC NWB pain (per 10 points)	0.7 [− 0.3;1.7]	0.18	–	–	–	–	0.2 [− 0.6;1.0]	0.62
Age (per year)	0.2 [− 0.2;0.6]	0.21	0.0 [− 0.2;0.2]	0.95	0.0 [− 1.4;1.4]	0.98	0.0 [− 0.2;0.2]	0.99
BMI (per 1 kg/m^2^)	− 1.4 [− 1.8;− 1.0]	*< 0.001*	− 1.4 [− 1.8;− 1.0]	*< 0.001*	− 1.4 [− 1.8;− 1.0]	*< 0.001*	− 1.5 [− 1.7;− 1.3]	*< 0.001*
Female sex	17.4 [12.3;22.5]	*< 0.001*	18.4 [13.9;22.9]	*< 0.001*	18.5 [10.0;23.0]	*< 0.001*	17.9 [13.6;22.2]	*< 0.001*
KL (per KL grade)	0.2 [− 1.8;2.2]	0.84	0.3 [− 1.7;2.3]	0.76	0.5 [− 1.5;2.5]	0.62	0.0 [− 2.0;2.0]	0.98

Effects of the baseline body mass index, sex, the sum of Kellgren-Lawrence grade of the non-target and the target knee and WOMAC pain to the baseline osteocalcin value, in percent and in univariate and multivariate analyses. WOMAC pain and sub-categories are calculated as the sum of both knees, normalised to 0–100 and analysed as the sum of the five pain sub-score questions, and as composites of weight-bearing (WB) pain or non-weight-bearing (NWB) pain. As the category “WOMAC pain” contains variables also included in “WOMAC WB pain” and “WOMAC NWB pain”, one multivariate model per each of these groups is performed. The parameter estimates and the standard error of estimate from the regression analyses are back-transformed in the presentation of the outcome of the regression analyses whereby the estimated effect and the confidence interval of the baseline characteristics will be given in the unit of percentage of the biomarker value. *p* values < 0.05 were considered significant and highlighted in italics. *WOMAC* Western Ontario and McMaster Universities Osteoarthritis Index, *BMI* body mass index, *KL* Kellgren-Lawrence

The biomarkers of bone turnover uCTX-I and s-osteocalcin were significantly yet modestly correlated with uCTX-II (correlation coefficient of 0.337 and 0.210, respectively). When analysing the markers for correlations within the group of females only, the correlation was slightly reduced, as the correlation between uCTX-I and s-osteocalcin and uCTX-II was 0.271 and 0.129, respectively. Similar findings were made between markers in males, where uCTX-I and s-osteocalcin were correlated to uCTX-II with coefficients of 0.236 and 0.119, respectively (all correlations *p* < 0.0001).

## Discussion

The current analyses are based on data from two major clinical trials in symptomatic knee OA involving 1241 patients and found that BMI, age, gender, the radiographic stage of knee OA and notably weight-bearing pain are independently and statistically significantly associated with uCTX-II.

The data suggest that unilateral radiographic knee OA is rare in a population of symptomatic knee OA, as only one patient with a KL score of 3 or above in one knee had a KL score of 0 in the contralateral knee, and very few (*n* = 19, 1.6%) had a KL score of 1 in the presence of KL 3 or above in the contralateral knee. Data available in the literature on the natural presence of unilateral OA indicates a much larger proportion of patients with unilateral OA than found in the current report [18, 19], yet the definition of unilateral OA in published reports varies. Presence of unilateral OA has been shown to influence patient-reported physical function impairment more than bilateral OA [18], and it has been speculated that the presence of bilateral OA may hamper joint-specific pain reporting, perhaps most importantly in clinical trials with localised treatment (topical or intra-articular). The nature of the current study as a randomised clinical trial may have hypothetically biased the population towards a bilateral one and thus may not be suitable for direct comparison with population studies, although no evidence of such bias was identified.

The lack of significant associations between uCTX-I, osteocalcin and radiographic disease stage suggest that these markers may not be sensitive measures of the role of bone turnover in the process of OA. The cross-sectional nature of the analysis, however, excludes the possibility of assessing a causal association between these bone turnover markers with disease progression. The findings partly corroborate previous reports from both smaller trials and larger cohort studies including the Osteoarthritis Initiative, in which serum CTX-I was not associated with radiographic disease of knee OA, while uCTX-II was [6, 8, 12].

In contrast to the bone turnover biomarkers mentioned above, the associations found between uCTX-II and knee OA radiographic severity may suggest that the release of the fragment occurs at a higher rate in late disease compared to early disease, but further longitudinal research is needed to evaluate this hypothesis. Secondly, it appears that the radiographic severity of the most affected knee joint may be responsible for the majority of uCTX-II release, which could be a potential confounder when assessing alterations of uCTX-II as a biomarker of OA disease modification in clinical trials, unless at least both knees are taken into account, although this question is also not possible to fully address in the current analysis. The hypothesis that the extent of cartilage degradation as reflected by uCTX-II is accelerated in late-stage OA, compared to early OA, also requires longitudinal data to be evaluated and was not assessed in this analysis. It has previously been reported that uCTX-II may reflect osteoclastic resorption of calcified cartilage, which is more abundant closer to the bone tidemark [15], and another report has localised the concentration of the uCTX-II neoepitope to be highest at the cartilage-bone interface [20]. This may explain the elevated levels of uCTX-II seen in late-stage subjects in the current report, as the uCTX-II fragment may flow more freely from existing cartilage in subjects with less cartilage, closer to deep layer of calcified cartilage compatible with KL grade 3 or 4 in one or both arthritic knees, rather than reflecting increased cartilage turnover at late stages of the disease. The Kellgren-Lawrence grading is based on radiographic assessments of structural features including osteophytes, bone sclerosis and joint-space narrowing; hence, the number and severity of osteophytes increase with the KL score. The finding that uCTX-II is associated with KL grade supports the report by van Spil et al. which evaluated patients from the Cohort Hip and Cohort Knee (CHECK) cohort with no or doubtful radiographic OA and found that uCTX-II, adjusted for other co-variates, was associated with the osteophyte area, although only in painful knees [16]. The biomarkers CTX-I and CTX-II have both previously been found to be associated with severity and extent of MRI bone marrow lesions in the data from the Osteoarthritis Initiative (OAI), in which CTX-II was also found to be associated with osteophytes [21] and in an earlier report also with reduced joint surface area [22]. Another report evaluating MRI and biomarkers from OAI did not find associations between femoral or tibial cartilage features and CTX-II, but found a weak association between cartilage T2 of the patellar region and CTX-II [23]. It is uncertain from the results of the current analysis if the association between CTX-II and KL grade is driven by osteophytes, joint-space narrowing or bone sclerosis. The current report corroborates the previously described significant correlations between known biomarkers of bone turnover and uCTX-II as reported by van Spil et al. [14]. Other literature reports suggest that CTX-II may arise from underlying similarly accelerated processes involving both cartilage and bone [14, 15], but the current report does not provide direct evidence of the tissue origin of the fragment. The analysis in the current report of correlations within each gender group does not indicate that the markers are differentially correlated in males vs. females.

Previous research has described that levels of uCTX-I and osteocalcin are inversely associated with BMI and positively associated with age and female gender [6, 14, 24], although a causal relationship has not been fully established. The current analysis did not find a significant association between age and these bone markers; however, that may be explained by the almost exclusively post-menopausal study population. Jørgensen and colleagues reported that levels of CTX-I appeared to be elevated in post-menopausal female subjects compared to pre-menopausal, but analysis per decade suggested that the elevation was not linear across the periods of pre- and post-menopause [24]. Previous reports also describe a relatively weak, positive association between the BMI and female gender, age and uCTX-II [6, 14]. The data in the current report supports these findings. The average level of uCTX-II in the groups of highest combined KL grades was found to be more than double that of those with early disease stages, suggesting that the majority of this elevation was not solely driven by factors associated with age, gender nor BMI, which was also confirmed in multivariate analyses as discussed below. Biomarker levels between male or female groups of identical KL scores were found to be higher in females compared to those in males in some but not all KL score groups. This finding adds to existing knowledge regarding the association of the markers and female gender in OA [6, 14], but warrants further research on the potential relevance of gender as an isolated factor for use of these biomarkers in OA in the context of structural features.

The results suggesting associations between CTX-II and pain support previous findings [10]. The limitations of the current analysis do not permit speculation regarding the causal nature of the association, but further research could be useful to evaluate if pain intensity may be directly influenced by processes leading to release of CTX-II, independently of the severity of radiographic disease. We found that the category of weight-bearing pain remained associated with CTX-II, while non-weight-bearing pain was not. This may suggest that non-weight-bearing pain is less associated with active biochemical processes involved in structural progression and perhaps influenced by extra-articular changes in the perception of pain, e.g. central sensitisation, but further studies with a more suitable design to investigate this matter are needed to support this hypothesis. A recent report found that pain at rest was more associated with features of neuropathic pain as compared to weight-bearing pain, which supports this hypothesis [25]. If this hypothesis is confirmed, it implies that the sensitivity of the WOMAC pain instrument may be improved in clinical trials evaluating the influence of an intervention targeting pathological degradation of joint tissues, in addition to analgesics acting locally, such as topical non-steroidal anti-inflammatory drugs, by focusing on the weight-bearing pain category instead of the total pain instrument. The current data did not indicate differences in the association between uCTX-II and pain intensity between genders.

Our data support prior results indicating that uCTX-II is an important biomarker in OA [6–8, 12], but suggest that uCTX-II has limited utility in assessing osteoarthritic characteristics solely originating from bone, regardless of aetiology.

### Limitations

According to Meulenbelt et al., OA in the knee, hip, facet and hand joints contributes independently to uCTX-II levels [13]. The results of the current analysis are limited by the lack of radiographic assessments of non-knee joints, potentially confounding the level of uCTX-II by the presence of underreported or unknown non-knee OA, which should be taken into consideration in the interpretation of the results. Further, as systemically circulating proteins, all the biomarkers discussed in this report may also originate from non-articular tissues in the body, and particularly CTX-I and osteocalcin are known to reflect the general bone turnover [26, 27]. As differences in bone turnover between the subjects of the current analysis may exist, particularly in the presence of bone diseases such as osteoporosis, but are not reported, it represents an unknown source of confounding to the results of particularly CTX-I and osteocalcin.

The comparison between levels of biomarkers in groups of KL grades in different genders results in a smaller sample size of some groups. While the presence of these groups do not substantially influence the conclusions of this report, the data of groups of males with KL grades 1–3, 2–4 and particularly 3–4 should be reviewed with caution, as these all were based on less than 10 observations per group.

As discussed above, as a cross-sectional analysis, the current report does not allow evaluations of causal relationships between biomarkers and other variables including the Kellgren-Lawrence grading scale, which itself is associated with limitations regarding intra- and inter-reader reliability.

## Conclusions

These results confirm that levels of uCTX-II are independently associated with radiographic severity of OA and pain intensity. CTX-II was associated with weight-bearing pain, but not non-weight-bearing pain, independent of BMI, age, gender and KL grade. Bilateral OA knee joints appear to contribute to uCTX-II levels in an incremental manner according to radiographic severity of single joints. The data suggest that biomarker differences between genders should be taken into account when evaluating these markers in the context of structural features of OA.

## Data Availability

The datasets generated and/or analysed during the current study are not publicly available.

## Abbreviations

ANOVA: Analysis of variance
BMI: Body mass index
CHECK: Cohort Hip and Cohort Knee
GCP: Good Clinical Practice
ICH: International Conference on Harmonisation
JSW: Joint space width
KL: Kellgren-Lawrence
OA: Osteoarthritis
uCTX-I: Urine C-telopeptide of cross-linked collagen type I
uCTX-II: Urine C-telopeptide of cross-linked collagen type II
WOMAC: Western Ontario and McMaster Universities Osteoarthritis Index
